# Molecular epidemiology and genotype distribution of Human Papillomavirus (HPV) among Arab women in the state of Qatar

**DOI:** 10.1186/s12967-014-0300-4

**Published:** 2014-11-26

**Authors:** Devendra Bansal, Asha A Elmi, Sini Skariah, Pascale Haddad, Laith J Abu-Raddad, Aysha H Al Hamadi, Nady Mohamed-Nady, Nahla M Affifi, Randa Ghedira, Elham Hassen, Asma AJ Al-Thani, Afaf AHM Al-Ansari, Ali A Sultan

**Affiliations:** Department of Microbiology and Immunology, Weill Cornell Medical College - Qatar, Cornell University, Qatar Foundation - Education City, Doha, Qatar; Infectious Disease Epidemiology Group, Weill Cornell Medical College - Qatar, Cornell University, Qatar Foundation - Education City, Doha, Qatar; Department of Obstetrics & Gynecology, Weill Cornell Medical College - Qatar, Cornell University, Qatar Foundation - Education City, Doha, Qatar; Department of laboratory Medicine and Pathology, Cytopathology, Hamad Medical Corporation, Doha, Qatar; Qatar Biobank, Qatar Foundation, Doha, Qatar; Laboratoire d’Immuno-Oncologie Moléculaire Faculté de Médecine de Monastir, 5019 Monastir, Tunisia; Health Sciences Department, Biomedical Sciences Program, University of Qatar, Doha, Qatar; Women’s Hospital, Hamad Medical Corporation, Doha, Qatar

**Keywords:** Human Papilloma virus, Qatar, Genotyping, Prevalence, Arab women, Cytology

## Abstract

**Background:**

Human Papilloma Virus (HPV) infection is the major cause of cervical cancer worldwide. With limited data available on HPV prevalence in the Arab countries, this study aimed to identify the prevalence and genotypic distribution of HPV in the State of Qatar.

**Methods:**

3008 cervical samples, exclusively of women with Arabic origin residing in Qatar were collected from the Women’s Hospital and Primary Health Care Corporation in Doha, State of Qatar. HPV DNA detection was done using GP5+/6+ primers based real time-polymerase chain reaction (RT-PCR) assay followed by the usage of HPV type specific primers based RT- PCR reactions and Sanger sequencing for genotype identification.

**Results:**

Similar prevalence rates of HPV infection was identified in both Qatari and non-Qatari women at 6.2% and 5.9% respectively. HPV prevalence rate of 5.8% and 18.4% was identified in women with normal cytology and in women with abnormal cytology respectively. HPV 81, 11 and 16, in decreasing order were the most commonly identified genotypes. HPV 81 was the most frequent low-risk genotype among women with both normal (74.0%) and abnormal (33.3%) cytology. HPV 16 (4.6%) was identified as the predominant high-risk HPV genotype among women with normal cytology and HPV 16, HPV 18, and HPV 56 (22.2% each) were the most common identified high-risk genotypes in women with abnormal cytology.

**Conclusions:**

The overall HPV prevalence in Arab women in Qatar was identified as 6.1% with an increased HPV prevalence seen in women with abnormal cytology results and no significant trends seen with age. In contrast to Western countries, we report a varied genotypic profile of HPV with a high prevalence of low-risk HPV genotype 81 among the Arab women residing in Qatar.

## Background

In developing countries, cervical cancer is the most common cancer among women with more than 85% of the global cervical cancer deaths occurring in these countries [1,2]. Molecular epidemiological studies have shown that Human Papillomavirus (HPV) infection represents the major etiological factor of cervical cancer [3,4]. To date, more than 200 HPV genotypes have been identified and characterized based on nucleotide sequence relatedness of the L1 gene which codes for the major HPV capsid protein [5]. Among these, based on their oncogenic potential via associations with cervical cancer and precancerous lesions, about 30 to 40 genotypes are divided into high-risk (HR) genotypes that cause cervical neoplasia, and low-risk (LR) HPV genotypes that cause mild dysplasia [5-7].

The prevalence of HPV infections in women within the general population differs considerably between countries and regions, as well as within regions, ranging from 1.6-41.9% [8]. Very limited data is available on the prevalence of HPV infection in the Arab countries where social, cultural and sexual behaviors may differ from the more-well reported Western countries [9,10]. Among the few published studies, HPV prevalence has been reported between 0% and 25% in women with normal cytology and up to 98% in women with abnormal cytology in the Extended Middle Eastern and North African (EMENA) countries [8,10-12]. Additionally, in a previous pilot study in Qatar, Al-Thani et al., identified a 64% prevalence of HPV in a cohort of women at high-risk for the infection [13].

Qatar is an Arab State located in Western Asia. Recent economic growth and globalization has resulted in a large influx of foreign expatriates from Western, other Middle Eastern, African and Asian countries leading to a dynamic socio-economic environment which possibly may affect social and sexual lifestyles in this country. However, there is limited data to investigate how these changes have affected the prevalence of sexually transmitted diseases such as HPV among the general population. Molecular epidemiological studies are warranted to understand the current disease burden posed by the circulating HPV genotypes and for predicting future projections of sexually transmitted diseases and cervical cancer in the population and within key sub-populations. Therefore, in the present study, we identified the prevalence and genotypic distribution of HPV among Arab women, with normal or abnormal cytology, living in the state of Qatar. The findings of this paper are critical towards estimation of relevance and outcome of HPV vaccination and HPV screening for cancer prevention in Qatar, both of which are not currently practiced in this country.

## Materials and methods

### Ethical considerations, study population and sample collection

This study was approved (Protocol no. - 10165/10) by the Institutional Review Board (IRB) of the Weill Cornell Medical College in Qatar (WCMCQ) and Hamad Medical Corporation (HMC) Research Office, Doha, Qatar. During March 2012-January 2013, a total of 3008 cervical samples, exclusively of women with Arabic origin (nationals of any of the 22 countries in the League of Arab States) residing in Qatar were collected from the women attending Women’s Hospital at HMC and Primary Health Care Corporation in Doha, Qatar for routine gynecological care/clinical symptoms. Inclusion criteria included married, non-pregnant women attending the above clinics. Only married or previously married women were considered in this study as Pap smear tests for never married women are not culturally acceptable. Women with known presence of cervical cancer and immunocompromised patients were excluded from this study. All samples fitting the inclusion criteria were included.

Cervical samples were collected in ThinPrep vials (BD SurePath™) for Pap smear assay and molecular HPV typing. ThinPrep cytological smears were screened at HMC and reported according to the Bethesda system for reporting of cervical cytology [14]. For each woman, other collated data include age, nationality, clinical history, and cytological diagnosis.

### DNA extraction and detection of HPV infection by real time PCR

Viral DNA from cervical samples was extracted by QIAamp MinElute virus spin kit according to the manufacturer’s instructions (Qiagen, CA, USA). To determine the presence of HPV-DNA, L1 consensus primers (GP5+: 5′- TTT GTT ACT GTG GTA GAT ACT AC -3′ and GP6+: 5′- GAA AAA TAA ACT GTA AAT CAT ATT C -3′) that amplify a conserved 150 bp sequence of the L1 open reading frame was used [15]. Human β-globin gene primers (PCO3: 5′- ACA CAA CTG TGT TCA CTA GC -3′ and PCO4: 5′- CAA CTT CAT CCA CGT TCA CC -3′) were used [15] to ensure the DNA quality of the samples.

Real-time polymerase chain reaction (RT-PCR) assay was carried out in ABI 7500 (Applied Biosystems, CA, USA) to detect HPV DNA using the following protocol: 2 μL extracted genomic DNA (5 ng/μL) was combined with 12.5 μl of 2X SyberGreen (Applied Biosystems, CA, USA) containing ROX as a passive reference along with 10 pmol (10 μM) each of forward and reverse primers (GP5+ and GP6+ or PCO3 and PCO4) and the mixture was made up to 25 μL volume with nuclease free water (Ambion, CA, USA). The PCR amplification was initiated at 95°C for 10 min and followed by 45 amplification cycles (denaturation at 95°C for 20 sec, annealing at 50°C for 30 sec and extension at 60°C for 30 sec).

All the samples were analyzed in duplicates on 96 well plates (Applied Biosystems, CA, USA). A positive control (plasmid carrying cloned HPV L1 fragment) and a negative control (nuclease free water) were included in each amplification reaction. HPV positive samples were detected by analyzing the amplicon dissociation curve and samples showing a melting temperature (Tm) between 75°C and 80°C were considered as positive. PCR products were also evaluated for β globin and L1 gene bands on 2% agarose gel stained with ethidium bromide and visualized with UV light.

### HPV genotyping by RT-PCR kits and DNA sequencing

Identification of the infecting HPV genotype(s) in the samples which tested positive for HPV DNA was done using an RT-PCR based kit (Sacace Biotechnology, Como, Italy), which tests for 12 high-risk HPV genotypes (16, 18, 31, 33, 35, 39, 45, 51, 52, 56, 58, and 59) and 2 low-risk HPV genotypes (6,11). The reactions were carried out according to the manufacturer’s instructions (Sacace Biotechnology, Como, Italy).

All samples, which tested positive for HPV infection by RT-PCR, but where genotyping could not be assigned on the basis of the typing method described above, were subsequently subjected to sequencing (Genewiz Inc., NJ, USA). PCR using GP5+/GP6+ primer set and same reaction conditions as used during detection of HPV infection in cervical samples was used to generate amplicons. In cases where the primary PCR yields were low, an additional round of PCR using 2 ul of the primary PCR product and the same primers was done to ensure enough yields to perform direct sequencing. The PCR products were purified using MinElute PCR Purification Kit according to manufacturer’s instructions (Qiagen, CA, USA). Purified products were sequenced using the Sanger method (Genewiz, NJ, USA) with GP6+ primer. The sequences were aligned using SeqMan Pro module of Lasergene 10.0 software (DNASTAR, WI, USA). For genotyping, 35 bp sequences adjacent to the GP5+ primer-binding site was used for nucleotide-nucleotide BLAST analysis (blastn) against known HPV genotype sequences in the GenBank database (www.ncbi.nlm.nih.gov/BLAST/). This 35 bp hyper-variable region of the L1 gene has been shown to be sufficient for distinguishing between various HPV genotypes [16]. Genotypes were assigned only when there was a 100% similarity between the 35 bp query and the subject sequence.

### Statistical analysis

**Figure 1:**
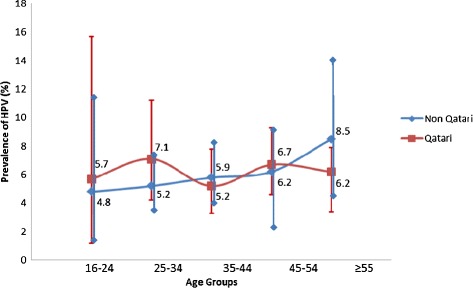
**Prevalence of HPV by age groups and nationality and their corresponding 95% confidence interval (CI) among Arab women in Qatar.**

Statistical analyses were performed using IBM SPSS version 21.0. Sample characteristics including age, cytology results, clinical findings and nationality were summarized using frequency distributions to generate the numbers and percentages (Table 1). The prevalence of HPV positivity by each of the above mentioned characteristics was assessed using the chi-squared test (Table 1). Unadjusted and adjusted odds ratios (ORs) for HPV positivity along with their 95% confidence intervals (CI) were calculated according to the selected characteristics summarized in Table 1, using bivariate and multivariable logistic regressions, respectively. The prevalence of HPV along with the 95% CI among Qatari and non-Qatari samples was plotted by age group (Figure 1). Chi-squared test for trend was used to assess whether there is an increasing or decreasing trend in the proportion of HPV positivity by age among Qatari and non-Qatari women. A *p*-value of less than 0.05 was considered significant. The distribution of HPV genotypes was summarized using frequency distribution and stratified by cytology results (abnormal versus normal cytology) (Table 2).

**Table 1:** **Unadjusted and adjusted odd ratios (ORs) for HPV positivity and their corresponding 95% confidence intervals (CIs) according to selected descriptive characteristics among 3008 women in Qatar**

	**Total no. women**	**HPV positive, n (%)**	**Unadjusted OR (95% CI)**	**Adjusted OR (95% CI)**
**Age groups**				
16-24	136 (4.5)	7 (5.1)	1	1
25-34	756 (25.1)	44 (5.8)	1.14 (0.50-2.58)	1.20 (0.53-2.73)
35-44	919 (30.6)	51 (5.5)	1.08 (0.48-2.44)	1.15 (0.51-2.59)
45-54	835 (27.8)	54 (6.5)	1.27 (0.57-2.86)	1.36 (0.60-3.07)
≥55	362 (12.0)	26 (7.2)	1.43 (0.60-3.37)	1.49 (0.63-3.54)
**Cytology results**				
Normal cytology	2959 (98.4)	173 (5.8)	1	1
Abnormal cytology	49 (1.6)	9 (18.4)	3.62 (1.73-7.59)*	3.68 (1.75-7.75)
**Clinical findings**				
Routine smear test	2401 (79.8)	142 (5.9)	1	1
Symptomatic results^≠^	607 (20.2)	40 (6.6)	1.12 (0.78-1.61)	1.15 (0.80-1.65)
**Nationality**				
Qatari	1404 (46.7)	87 (6.2)	1	1
Non-Qatari	1604 (53.3)	95 (5.9)	0.95 (0.71-1.29)	1.00 (0.73-1.36)
**Nationality**				
Qatari	1404 (46.7)	87 (6.2)	1	--
Egyptian	465 (15.5)	26 (5.6)	0.90 (0.57-1.41)	
Arabian peninsula^$^	310 (10.3)	14 (4.5)	0.72 (0.40-1.28)	
Fertile Crescent^£^	566 (18.8)	37 (6.5)	1.06 (0.71-1.58)	
North Africa^#^	95 (3.2)	7 (7.4)	1.20 (0.54-2.68)	
East Africa^^^	168 (5.6)	11 (6.5)	1.06 (0.55-2.03)	

*p-value < 0.05.

^$^Arabian Peninsula excluding Qatar (KSA, Kuwait, Bahrain, UAE, Oman, Yemen).

^£^Fertile Crescent excluding Egypt (Iraq, Jordan, Lebanon, Palestine, Syria).

^#^North Africa (Morocco, Algeria, Tunisia, Libya and Mauritania).

^^^East Africa (Somalia, Djibouti, Sudan and Comoros).

^≠^Symptomatic results include: cervical erosion, genital warts, lower abdominal pain, menorrhagia, pelvic pain, post coital bleeding, primary infertility, secondary infertility, vaginal bleeding, vaginal discharge, vaginal spotting and vulva itching.

**Table 2:** **The distribution of HPV types among HPV DNA positive cervical samples among Arab women in Qatar**

	**General Arab population in Qatar**													
	**Normal cytology (n = 2959)**						**Abnormal cytology (n = 49)**						**All (n = 3008)**	
**HPV type**	**Single**	**Double**	**Multiple**	**Unknown**	**Total**	**Percent**	**Single**	**Double**	**Multiple**	**Unknown**	**Total**	**Percent**	**Total**	**Percent**
HPV positive	144	19	5	5	173	5.8	5	3	1	0	9	18.4	182	6.1
High-risk														
16	4	2	2	--	8	4.62	0	1	1	--	2	22.22	10	5.49
18	0	0	3	--	3	1.73	0	2	0	--	2	22.22	5	2.74
31	1	2	1	--	4	2.31	0	1	0	--	1	11.11	5	2.74
33	1	2	1	--	4	2.31	0	0	0	--	0	0.00	4	2.19
35	0	1	1	--	2	1.15	0	0	0	--	0	0.00	2	1.09
39	0	1	1	--	2	1.15	0	0	0	--	0	0.00	2	1.09
45	2	3	0	--	5	2.89	0	0	0	--	0	0.00	5	2.74
51	0	1	1	--	2	1.15	0	0	1	--	1	11.11	3	1.64
52	1	0	0	--	1	0.57	0	0	0	--	0	0.00	1	0.54
56	0	4	1	--	5	2.89	1	1	0	--	2	22.22	7	3.84
58	1	0	1	--	2	1.15	1	0	0	--	1	11.11	3	1.64
59	4	1	1	--	6	3.46	0	0	0	--	0	0.00	6	3.29
Intermediate risk														
67	4	1	1	--	6	3.46	0	0	0	--	0	0.00	6	3.29
Low-risk														
6	1	3	0	--	4	2.31	0	0	0	--	0	0.00	4	2.19
11	4	5	2	--	11	6.35	0	1	1	--	2	22.22	13	7.14
81	119	9	0	--	128	73.98	3	0	0	--	3	33.33	131	71.97
90	2	3	0	--	5	2.89	0	0	0	--	0	0.00	5	2.74

*Note:* Abnormal cytology includes LGSIL + HGSIL.

## Results

### Demographic and clinical characteristics of the study population

Samples were collected from women visiting hospital/clinic for routine gynecological care or for different clinical symptoms such as vaginal discharge, vaginal bleeding, genital warts, vulva itching, lower abdominal & pelvic pain, infertility, menorrhagia, among others. The population cohort analyzed in this study was recruited to reflect the general Arab women population in Qatar. This is supported by the clinical findings for subjects summarized in Table 1, which shows that 79.8% (n = 2401) of the women underwent routine gynecologic care and only 20.2% (n = 607) were symptomatic. Additionally, based on Pap smear test, 98.4% (n = 2959) of the women had normal cytology results without any lesions. A minority of women (n = 49, 1.6%) had abnormal cytology results with either low-grade squamous intraepithelial lesion (LGSIL) indicating mild dysplasia or high-grade squamous intraepithelial lesion (HGSIL) indicating severe intraepithelial neoplasia.

All age groups were well represented as seen in Table 1. The age range across the sample was 16–84 years with a mean age of 41.6 years (SD = 10.9). 46.7% (n = 1404) of the women in the sample were Qataris with the rest belonging to other Middle Eastern, North and East African countries in the League of Arab States (Table 1), which is reflective of the make-up of the general Arab women population in Qatar.

### HPV DNA prevalence in Arab women

The overall HPV prevalence in Arab women in Qatar was 6.1% (n = 182). Similar prevalence rates of HPV infection was identified in both Qatari and non Qatari women at 6.2% (n = 87, 95% CI: 4.9-7.5%) and 5.9% (n = 95, 95% CI: 4.8-7.1%) respectively (Table 1). Among the non-Qatari Arab women, HPV prevalence was highest in women originating from African and fertile crescent countries (North Africa: 7.4% (n = 7, 95% CI: 2.0-12.7%), Fertile Crescent excluding Egypt: 6.5% (n = 37, 95% CI: 4.5-8.6%) and East Africa: 6.5% (n = 11, 95% CI: 2.8-10.3%)) followed by a 5.6% rate in Egyptian women and (n = 26, 95% CI: 3.5-7.7%) and a lower prevalence of 4.5% (n = 14, 95% CI: 2.2-6.8%) in women from the Arabian peninsula excluding Qatar (Table 1).

HPV prevalence was identified as 5.8% (n = 173, 95% CI: 5.0-6.7%) among women with normal cytology. Among women with abnormal cytology, the overall HPV prevalence was detected as 18.4% (n = 9, 95% CI: 7.1-29.6%) (Table 1) which included 36 samples with LGSIL of which, 8 were HPV positive and 13 samples with HGSIL of which, 1 was positive for HPV. This increased association between HPV prevalence and abnormal cytology results was also found to be statistically significant (OR: 3.68, 95% CI: 1.75-7.75%). However, no significant difference was observed in HPV prevalence in women coming for routine care vs with specific clinical complaints (OR: 1.15, 95% CI: 0.80-1.65%).

HPV prevalence overall was found to be highest (7.2%) in the age group >55 (n = 26, 95% CI: 0.63-3.54%) (Table 1). Measured HPV prevalence was highest in the 25-34 age group (n = 17, 7.1%, 95% CI: 3.8-10.4%) among Qatari women, and in the age group >55 (n = 13, 8.5%, 95% CI: 4.0-13.0%) among non-Qatari women (Table 1). HPV prevalence appeared to increase slightly with age in non-Qatari women, while no such trend was seen in Qatari women (Figure 1). However, by chi-squared trend analyses, no significant trends were found in HPV prevalence versus age for either Qatari women or non-Qatari women.

### Distribution of HPV genotypes in Arab women

The 182 cervical samples, which were identified as positive for HPV DNA, were further analyzed to identify the infecting HPV genotype and were classified based on their oncogenic potential as shown in Table 2. Among HPV positive women, taking into account the total frequency of occurrence of each specific genotype occurring either as single/multiple infection *(sum of frequency of genotypes belonging to a specific risk category/total number of women (182))*, 84.0% (n = 153, 95% CI: 76.9-88.3%) had at least one low-risk HPV genotype, 3.3% (n = 6, 95% CI: 1.2-7.0%) had one intermediate-risk HPV genotype, and 29.1% (n = 53, 95% CI: 22.6-36.3%) had at least one high-risk HPV genotype (Table 2). Five samples (2.7%; 95% CI: 0.9-6.3%) remained uncharacterized and have been listed in Table 2 as unknown.

HPV 81 was the most frequent low-risk genotype among women with both normal (n = 128, 74.0%; 95% CI: 66.8-80.4%) and abnormal (n = 3, 33.3%; 95% CI: 7.5-70.1%) cytology (Table 2). Women with HPV 81 genotype were at significantly less risk of showing abnormal cytology than women with high-risk genotypes (OR: 0.13, 95% CI: 0.03-0.55), as described in Table 3. The only intermediate-risk HPV genotype identified was HPV 67, which had a 3.5% (n = 6, 95% CI: 1.3-7.4%) prevalence among women with normal cytology (Table 2). No cases of HPV 67 were identified among women with abnormal cytology. Meanwhile, HPV 16 (n = 8, 4.6%; 95% CI: 2.0-8.9%) was identified as the predominant high-risk HPV genotype among women with normal cytology, followed by HPV 59 (n = 6, 3.5%; 95% CI: 1.3-7.4%), HPV 56 and HPV 45 (n = 5, (2.9%, 95% CI: 0.9-6.6%) for each) (Table 2). For women with abnormal cytology, the most common identified high-risk genotypes were HPV 16, HPV 18, and HPV 56 (Table 2). For each of these genotypes, two cases were identified (22.2% (95% CI: 2.8-60.0%) for each).

**Table 3:** **Odd ratios (ORs) for abnormal cytology and their corresponding 95% confidence intervals (CIs) in HPV positive women according to genotype classification**

	**Total HPV positive women n (%)**	**Abnormal cytology n (%)**	**OR (95% CI)**
**Genotype classification** ^**£**^			
High risk	37 (20.3)	6 (16.2)	1
Intermediate risk	5 (2.8)	0	--
Low risk	13 (7.1)	0	--
Type 81	122 (67.0)	3 (2.5)	0.13 (0.03-0.55)*
Unknown	5 (2.8)	0	--

*p-value < 0.05.

^£^In case of mixed HPV infection where the infecting genotypes belong to different groups based on the above listed genotype classification categories, infected women were placed in the category corresponding to the infecting HPV genotype with the highest risk potential.

Single infection, double infections (infection with two different HPV genotypes), and multiple infections (infection with >2 different HPV genotypes) were noted. Among women with normal cytology, 144 (83.2%; 95% CI: 76.8-88.5%) had single infection, 19 (11.0%; 95% CI: 6.7-16.6%) had double infections, and 5 (2.9%; 95% CI: 0.9-6.6%) had multiple infections (Table 2). However, in women with abnormal cytology, only 5 (55.6%; 95% CI: 21.2-86.3%) had single infection, 3 (33.3%; 95% CI: 7.5-70.1%) had double infections, and 1 (11.1%; 95% CI: 0.3-48.2%) had multiple infections (Table 2).

## Discussion

HPV is one of the most infectious and widespread sexually transmitted diseases [17]. Nonetheless, scientific research on HPV and other sexually transmitted infections, despite recent progress, remains rather limited in EMENA, and is confronted with multiple challenges [18]. This is to our knowledge the largest cross-sectional survey describing the prevalence of HPV infection and distribution of HPV genotypes among Arab women from across the Arab World, but all of whom are residing currently in the State of Qatar.

With the advent of molecular tools, epidemiological studies are revealing the extent of HPV infection burden in different countries and among various clinical, ethnic, and risk populations [19-25]. The strength of the present study lies in the use of a standardized and sensitive molecular assay for HPV detection, rendering our findings amenable to a detailed analysis of HPV prevalence and distribution among general population of Arab women, and comparison to global patterns. Such analysis is relevant for planning of health service provision and development of appropriate interventions based on the current characteristics of the infection burden and implied future trends for squamous intraepithelial lesions and cervical cancer. The indications that HPV infection burden might be increasing in EMENA [10], adds further importance to this investigation.

In our study, HPV prevalence in Arab women with normal cytology was 5.8% which is comparable to state of the art studies on representative populations in EMENA [26-28], but overall substantially lower than other studies from different EMENA countries on different convenient and often not large samples [8,10,12]. Methodological differences probably explain the differences in the measured prevalence levels, especially so in relation to the representativeness of the tested populations. Though not based on a probability-based sample, the large sample size in our study, and the diverse profile of participants, facilitated a representation of the general population of Arab women residing in Qatar. Just as in global trends [29,30], we documented a large HPV prevalence of 18.4% in women with cervical abnormalities, however this prevalence rate is lower when compared to other published studies [29,31,32]. The reasons for this deviation are unclear and may be attributed to variations in the quality of the specimens tested and the sensitivity of the HPV detection assay used in this study [31].

The relatively low prevalence of HPV infection found in our study is consistent with the low incidence of cervical cancer in Qatar and EMENA, the lowest globally [10,12,33]. This is also supported by the conservative socio-cultural norms towards sexuality and the apparently sparse and poorly connected nature of sexual networks in this part of the world [10]. The nearly universal coverage of male circumcision in EMENA [10], with its protective effect against HPV infection [34], may have also contributed to a lower force of infection in the population and reduced risk for women to acquire HPV.

However, the observed levels of prevalence suggest that there are pockets of risks for acquiring the infection through sexual networks that are sufficiently conducive for sustainable propagation of this infection. These networks and exposure to the infection may not necessarily occur in Qatar. However with the high mobility of the population, where more than 75% of the population are expatriates [35], and where the population travels frequently, the infection transmission networks are probably intersecting with sexual networks in other countries. Based on the overall pattern of sexually transmitted infections in EMENA [10,36], it is likely also that infected women have acquired the infection from their spouses.

The age specific distribution of HPV prevalence in the present study shows remarkably a largely flat distribution across the age groups. This contrasts with the common age-specific distribution with a sharp peak in HPV prevalence among young women following their sexual debut [37]. However, this result affirms the flat distribution seen recently in quality HPV prevalence studies in EMENA [26-28]. While it is not clear why HPV prevalence age distribution differs in this part of the world, the prevailing sexually conservative norms, among other factors, may have driven differences in sexual networking leading to different infection age distribution.

We found a heterogeneous distribution of HPV genotypes, possibly due to broad geographical and cultural range of the nationalities included in this survey. In the present study, HPV16 was the most common genotype followed by HPV 56, 59, 18, 45, 31, 33, 51, 58, 35, 39 and 52 among the high-risk HPV group, HPV 67 in intermediate risk group and HPV 81,11 and 6 in the low-risk group. This is in agreement with global studies where HPV 16 was identified as the most common high-risk HPV genotype [38-40]. Several studies show that HPV 16, 18, 45, 31, 33, 52, 58, and 35, all of which were identified in Qatar in the present study may account for as much as 95% of cervical cancers [4,11,31,41]. In our study, high-risk HPV genotypes were found in both women with normal and abnormal cytology. HPV16 was the most common high-risk genotype (4.6%) among women with normal cytology and HPV 16 and 18 were the most common genotypes among women with abnormal cytology. When taken into consideration the fact that HPV 16 and 18, taken together, are responsible for about 70% of the cervical cancer cases worldwide [4,31,41], these results suggest the need for HPV screening even in absence of cytological abnormalities and indicate the inadequacy of cytology based assays for cancer screening.

In this study, HPV 81 was the most frequent low-risk genotype among women with both normal (74%) and abnormal (33.3%) cytology. This high HPV genotype 81 prevalence found in our study is unexpected. Though seen in a previous study in Southern China [42], it is possibly attributed to variations in the geographic distribution of HPV genotypes and the sensitivities of different methods used for HPV detection [42]. HPV 81 is classified as a low-risk genotype and has not been reported in high numbers in worldwide population studies, however it is one of the most frequently observed HPV genotypes in HIV-positive patients and is also reported to be associated with precancerous or cancerous lesions [32,42-46]. We also found an intratypic variant of HPV 81, which matched a unique isolate identified in an HIV-1 positive patient in India [47]. However, in this study, women with HPV 81 genotype had a significantly reduced risk of showing abnormal cytology than women with high-risk genotypes, which further enforces the low-risk classification of this HPV genotype.

In the present study, women with normal cytology mostly had a single HPV type infection (83.2%) but in patients with cytological abnormalities, infection with double and multiple HPV types were observed frequently (44.4%), which is similar with previous reports where multiple HPV infection with high-risk genotypes have found to be associated with a significantly increased risk of cervical intraepithelial neoplasia compared to infection with a single HPV type [48,49].

In this study, we recruited women attending hospitals for routine gynecological care/ clinical symptoms while excluding known cervical cancer patients to analyze HPV prevalence across a diverse range of women, however as all these women were patients, this population may not be completely representative of the general women population in Qatar. With only married/previously married women being included and no sexual behavior measures being collected as part of the study, due to the sensitivity of sexual behavior in this region, we were unable to examine specific risk factors for the infection. Another complication is the high rates of intermarriage between different nationalities and change in the nationality status of the women after marriage, which may bias the nationality specific HPV prevalence in this study.

## Conclusion

In conclusion, our study showed a relatively low prevalence (6.1%) of HPV infection in comparison to Western countries and presence of a varied genotypic profile of HPV with a high prevalence of low-risk HPV genotype 81 among general population of Arab women residing in Qatar. Though lower than in other countries and regions, our study suggests that there is sufficient infection burden to warrant public health interventions. HPV vaccination should be considered as a prevention intervention for girls as well as women in Qatar. The low levels of Pap smear screening in Qatar [50], just as in most EMENA countries [10,51], adds another reason to for HPV vaccination programs. This is also supported by a positive attitude towards HPV vaccination in at least few Muslim societies [52]. Though a cost-effectiveness analysis is yet to be conducted, based on analyses in other countries with some similarity in context [53,54], vaccination may offer a cost-effective method to reduce cervical cancer and its mortality. When considering a prophylactic vaccine, the data from this study show that the currently used quadrivalent vaccine “GARDASIL” (Merck & Co., Inc. USA) has the potential to prevent HPV infections in Arab women. However, as differences were noted in our study population with a high prevalence of HPV 81 and other high-risk/intermediate risk HPV genotypes types such as HPV 56, 59, 45 and 67, the potential for cancer prevention in this region may rise if these frequent high and intermediate risk HPV genotypes, and possibly HPV 81, are also included in future HPV vaccines targeting this specific population.

## Abbreviations

HPV: Human Papilloma Virus
EMENA: Extended Middle East and North African countries
IRB: Institutional Review Board
WCMCQ: Weill Cornell Medical College in Qatar
HMC: Hamad Medical Corporation
LR: Low-risk
HR: High-risk
HGSIL: High-Grade Squamous Intraepithelial Lesion
LGSIL: Low-Grade Squamous Intraepithelial Lesion

## References

[1] Denny L, Falk SJ, Section Editor Barbara Goff, Deputy Editor (2014). Screening for Cervical Cancer in Resource-Limited Settings. UpToDate.

[2] Jemal A, Bray F, Center MM, Ferlay J, Ward E, Forman D (2011). Global cancer statistics. CA Cancer J Clin.

[3] IARC (1995). Human Papillomaviruses. Human Papillomaviruses.

[4] Walboomers JMJM, Manos MM, Bosch FX, Kummer JA, Shah KV, Snijders PJ, Peto J, Meijer CJ, Muñoz N (1999). Human papillomavirus is a necessary cause of invasive cervical cancer worldwide. J Pathol.

[5] Munoz N, Bosch FX, de Sanjose S, Herrero R, Castellsague X, Shah KV, Snijders PJ, Meijer CJ (2003). Epidemiologic classification of human papillomavirus types associated with cervical cancer. N Engl J Med.

[6] Terai M, Burk RD (2002). Identification and characterization of 3 novel genital human papillomaviruses by overlapping polymerase chain reaction: candHPV89, candHPV90, and candHPV91. J Infect Dis.

[7] Munoz N, Castellsague X, de Gonzalez AB, Gissmann L (2006). HPV in the etiology of human cancer. Vaccine.

[8] Seoud M (2012). Burden of human papillomavirus-related cervical disease in the extended middle East and north Africa-a comprehensive literature review. J Low Genit Tract Dis.

[9] Wellings K, Collumbien M, Slaymaker E, Singh S, Hodges Z, Patel D, Bajos N (2006). Sexual behaviour in context: a global perspective. Lancet.

[10] Abu-Raddad L, Akala FA, Semini I, Riedner G, Wilson D, Tawil O (2010). Characterizing the HIV/AIDS Epidemic in the Middle East and North Africa: Time for Strategic Action. World Bank/UNAIDS/WHO Publication.

[11] Kahn JA (2009). HPV vaccination for the prevention of cervical intraepithelial neoplasia. N Engl J Med.

[12] Vaccarella S, Bruni L, Seoud M (2013). Burden of human papillomavirus infections and related diseases in the extended Middle East and North Africa region. Vaccine.

[13] Asma AJ, Al-Thani AIA-R, Afaf A-A, Mandy A, Moza A-K, Sabah A-L (2010). Prevalence of human papillomavirus infection in women attending a gynecology/oncology clinic in Qatar. Future Virol.

[14] Solomon D, Davey D, Kurman R, Moriarty A, O’Connor D, Prey M, Raab S, Sherman M, Wilbur D, Wright T, Young N, Forum Group Members-Bethesda 2001 Workshop (2002). The 2001 Bethesda System: terminology for reporting results of cervical cytology. J Am Med Assoc.

[15] de Roda Husman AM WJ, van den Brule AJ, Meijer CJ, Snijders PJ (1995). The use of general primers GP5 and GP6 elongated at their 3′ ends with adjacent highly conserved sequences improves human papillomavirus detection by PCR. J Gen Virol.

[16] Feoli-Fonseca JC, Oligny LL, Filion M, Brochu P, Simard P, Russo PA, Yotov WV (1998). A two-tier polymerase chain reaction direct sequencing method for detecting and typing human papillomaviruses in pathological specimens. Diagn Mol Pathol.

[17] Hariri SDE, Saraiya M, Unger E, Markowitz L (2011). Human Papillomavirus. VPD Surveillance Manual.

[18] Abu-Raddad LJ, Ghanem KG, Feizzadeh A, Setayesh H, Calleja JM, Riedner G (2013). HIV and other sexually transmitted infection research in the Middle East and North Africa: promising progress?. Sex Transm Infect.

[19] Burd EM (2003). Human papillomavirus and cervical cancer. Clin Microbiol Rev.

[20] Chen YC, Hunter DJ (2005). Molecular epidemiology of cancer. CA Cancer J Clin.

[21] Molijn A, Kleter B, Quint W, van Doorn LJ (2005). Molecular diagnosis of human papillomavirus (HPV) infections. J Clin Virol.

[22] Hoory T, Monie A, Gravitt P, Wu TC (2008). Molecular epidemiology of human papillomavirus. J Formos Med Assoc.

[23] Ashley Arney KMB (2010). Molecular Diagnostics of Human Papillomavirus. LabMedicine.

[24] Sait KH, Gazzaz FS (2011). Molecular tests to detect human papillomavirus infection in patients with cervical dysplasia and invasive cervical cancer in Saudi Arabia. Pathol Lab Med Int.

[25] Othman N, Othman NH (2014). Detection of human papillomavirus DNA in routine cervical scraping samples: use for a national cervical cancer screening program in a developing nation. Asian Pac J Cancer Prev.

[26] Hammouda D, Clifford GM, Pallardy S, Ayyach G, Chekiri A, Boudrich A, Snijders PJ, van Kemenade FJ, Meijer CJ, Bouhadef A, Zitouni Z, Habib D, Ikezaren N, Franceschi S (2011). Human papillomavirus infection in a population-based sample of women in Algiers, Algeria. Int J Cancer.

[27] Khodakarami N, Clifford GM, Yavari P, Farzaneh F, Salehpour S, Broutet N, Bathija H, Heideman DA, van Kemenade FJ, Meijer CJ, Hosseini SJ, Franceschi S (2012). Human papillomavirus infection in women with and without cervical cancer in Tehran, Iran. Int J Cancer.

[28] Raza SA, Franceschi S, Pallardy S, Malik FR, Avan BI, Zafar A, Ali SH, Pervez S, Serajuddaula S, Snijders PJ, van Kemenade FJ, Meijer CJ, Shershah S, Clifford GM (2010). Human papillomavirus infection in women with and without cervical cancer in Karachi, Pakistan. Br J Cancer.

[29] Munoz N (2000). Human papillomavirus and cancer: the epidemiological evidence. J Clin Virol.

[30] IARC (2005). Cervix Cancer Screening.

[31] Bosch FX, Burchell AN, Schiffman M, Giuliano AR, de Sanjose S, Bruni L, Tortolero-Luna G, Kjaer SK, Munoz N (2008). Epidemiology and natural history of human papillomavirus infections and type-specific implications in cervical neoplasia. Vaccine.

[32] Al-Awadhi RCW, Jaragh M, Al-Shaheen A, Sharma P, Kapila K (2013). Distribution of human papillomavirus among women with abnormal cervical cytology in Kuwait. Diagn Cytopathol.

[33] Drain PK, Holmes KK, Hughes JP, Koutsky LA (2002). Determinants of cervical cancer rates in developing countries. Int J Cancer.

[34] Larke N, Thomas SL, Dos Santos SI, Weiss HA (2011). Male circumcision and human papillomavirus infection in men: a systematic review and meta-analysis. J Infect Dis.

[35] World Migration 2005 (2005). Costs and Benefits of International Migration.

[36] Abu-Raddad LJ, Hilmi N, Mumtaz G, Benkirane M, Akala FA, Riedner G, Tawil O, Wilson D (2010). Epidemiology of HIV infection in the Middle East and North Africa. AIDS.

[37] Schiffman M, Castle PE (2005). The promise of global cervical-cancer prevention. N Engl J Med.

[38] Jacobs MV, Walboomers JM, Snijders PJ, Voorhorst FJ, Verheijen RH, Fransen-Daalmeijer N, Meijer CJ (2000). Distribution of 37 mucosotropic HPV types in women with cytologically normal cervical smears: the age-related patterns for high-risk and low-risk types. Int J Cancer.

[39] Rolon PA, Smith JS, Munoz N, Klug SJ, Herrero R, Bosch X, Llamosas F, Meijer CJ, Walboomers JM (2000). Human papillomavirus infection and invasive cervical cancer in Paraguay. Int J Cancer.

[40] Berumen J, Ordonez RM, Lazcano E, Salmeron J, Galvan SC, Estrada RA, Yunes E, Garcia-Carranca A, Gonzalez-Lira G, Madrigal-de la Campa A (2001). Asian-American variants of human papillomavirus 16 and risk for cervical cancer: a case-control study. J Natl Cancer Inst.

[41] Usubutun A, Alemany L, Kucukali T, Ayhan A, Yuce K, de Sanjose S, Font R, Lloveras B, Klaustermeier J, Quint W, Muñoz N, Bosch FX (2009). Human papillomavirus types in invasive cervical cancer specimens from Turkey. Int J Gynecol Pathol.

[42] Ngai Na Chloe Co L-OC, Joseph KF C, Joseph WO T, Enders KO N: **HPV prevalence and detection of rare HPV genotypes in Hong Kong women from Southern China with cytological abnormalities.** *International Scholarly Research Notices Virology* 2013, **2013**.

[43] Cerqueira DM, de SMD, Camara GN, Amaral FA, Oyama CN, dos Santos MQ, Martins CR (2007). High HPV genetic diversity in women infected with HIV-1 in Brazil. Arch Virol.

[44] Tornesello ML, Duraturo ML, Giorgi-Rossi P, Sansone M, Piccoli R, Buonaguro L, Buonaguro FM (2008). Human papillomavirus (HPV) genotypes and HPV16 variants in human immunodeficiency virus-positive Italian women. J Gen Virol.

[45] Garbuglia AR, Piselli P, Lapa D, Sias C, Del Nonno F, Baiocchini A, Cimaglia C, Agresta A, Capobianchi MR (2012). Frequency and multiplicity of human papillomavirus infection in HIV-1 positive women in Italy. J Clin Virol.

[46] Choi YDHC, Chung WJ, Jung WW, Lee JS, Nam JH, Lee MC, Juhng SW, Choi HS, Park CS (2009). Analysis of HPV-other samples by performing HPV DNA sequencing. Korean J Pathol.

[47] Mullick R SS, Chakrabarti S (2012). Genotypic distribution of different variants of oncogenic Human Papilloma Virus (HPV) among the sexually active HIV-1 positive population from Kolkata and Manipur. World J AIDS.

[48] Spinillo A, Dal Bello B, Gardella B, Roccio M, Dacco MD, Silini EM (2009). Multiple human papillomavirus infection and high grade cervical intraepithelial neoplasia among women with cytological diagnosis of atypical squamous cells of undetermined significance or low grade squamous intraepithelial lesions. Gynecol Oncol.

[49] Trottier H, Mahmud S, Costa MC, Sobrinho JP, Duarte-Franco E, Rohan TE, Ferenczy A, Villa LL, Franco EL (2006). Human papillomavirus infections with multiple types and risk of cervical neoplasia. Cancer Epidemiol Biomark Prev.

[50] Al-Thani A, Abdul-Rahim H, Alabsi E, Bsaisu HN, Haddad P, Mumtaz GR, Abu-Raddad LJ (2013). Prevalence of Chlamydia trachomatis infection in the general population of women in Qatar. Sex Transm Infect.

[51] Sancho-Garnier H, Khazraji YC, Cherif MH, Mahnane A, Hsairi M, El Shalakamy A, Osgul N, Tuncer M, Jumaan AO, Seoud M (2013). Overview of cervical cancer screening practices in the extended Middle East and North Africa countries. Vaccine.

[52] Baykal C, Al A, Ugur MG, Cetinkaya N, Attar R, Arioglu P (2008). Knowledge and interest of Turkish women about cervical cancer and HPV vaccine. Eur J Gynaecol Oncol.

[53] Human papillomavirus and HPV Vaccines (2007). Technical Information for Policy-Makers and Health Professionals.

[54] Ginsberg GM, Fisher M, Ben-Shahar I, Bornstein J (2007). Cost-utility analysis of vaccination against HPV in Israel. Vaccine.

